# Case Report: A rare case of primary undifferentiated pleomorphic sarcoma of the renal pelvis with high PD-L1 expression and a misleading positive urine FISH

**DOI:** 10.3389/fimmu.2026.1769769

**Published:** 2026-03-03

**Authors:** Jiameng Li, Xiaoyu Wang, Junhui Zhen, Mei Qi

**Affiliations:** 1 Department of Pathology, Qilu Hospital of Shandong University, Jinan, Shandong, China; 2 Department of Pathology, School of Basic Medical Sciences, Shandong University, Jinan, Shandong, China

**Keywords:** fluorescence *in situ* hybridization (FISH), pleomorphic undifferentiated sarcoma (UPS), programmed death receptor ligand 1 (PD-L1), renal pelvis, TP53

## Abstract

Primary undifferentiated pleomorphic sarcoma (UPS) of the renal pelvis is an extremely rare and aggressive mesenchymal malignancy. The diagnosis remains challenged, and its clinical characteristics and treatment strategies remain unclear. Urine fluorescence *in situ* hybridization (FISH) is a highly sensitive tool for detecting urothelial carcinoma (UC), but ‘false’-positive results can be observed in non-UC malignancies. We present a challenging case of primary UPS of renal pelvis with positive urine FISH and high programmed death receptor ligand 1 (PD-L1) expression, which complicates the initial diagnosis and opens avenues for potential immunotherapy.

## Introduction

The most common malignant tumor of the kidney is clear cell renal cell carcinoma, while primary renal sarcoma represents a rare entity, comprising only approximately 1-3% of all malignant renal neoplasms (1). Common histological subtypes include leiomyosarcoma, clear cell sarcoma, and liposarcoma (2). Undifferentiated pleomorphic sarcoma (UPS), formerly termed malignant fibrous histiocytoma, is a rare, highly malignant mesenchymal tumor. Its clinicopathological characteristic and treatment remains poorly understood (3). Although UPS mostly commonly occurs in the extremities and retroperitoneum, its presentation in the renal pelvis is exceedingly rare. This unusual location coupled with its nonspecific clinical presentation and aggressive biological behavior, poses unique diagnostic and therapeutic challenges. This case concerns a 75-year-old female patient who presented with persistent gross hematuria and renal pelvic mass, initially suspected to be urothelial carcinoma (UC). The diagnosis was subsequently revised to UPS based on comprehensive histopathological, immunohistochemical and next-generation sequencing (NGS) analyses. Notably, the combination of a positive urine fluorescence *in situ* hybridization (FISH) test and programmed death receptor ligand 1 (PD-L1) overexpression is highly atypical for sarcoma of the renal pelvis, underscoring the necessity of a multidisciplinary collaboration for accurate diagnosis and management.

This case report aims to elucidate the clinicopathological and molecular features of primary UPS of the renal pelvis. In the absence of established guidelines, this work intends to enrich the evidence base for this rare disease and to guide future clinical practice.

## Case presentation

**Figure 1:**
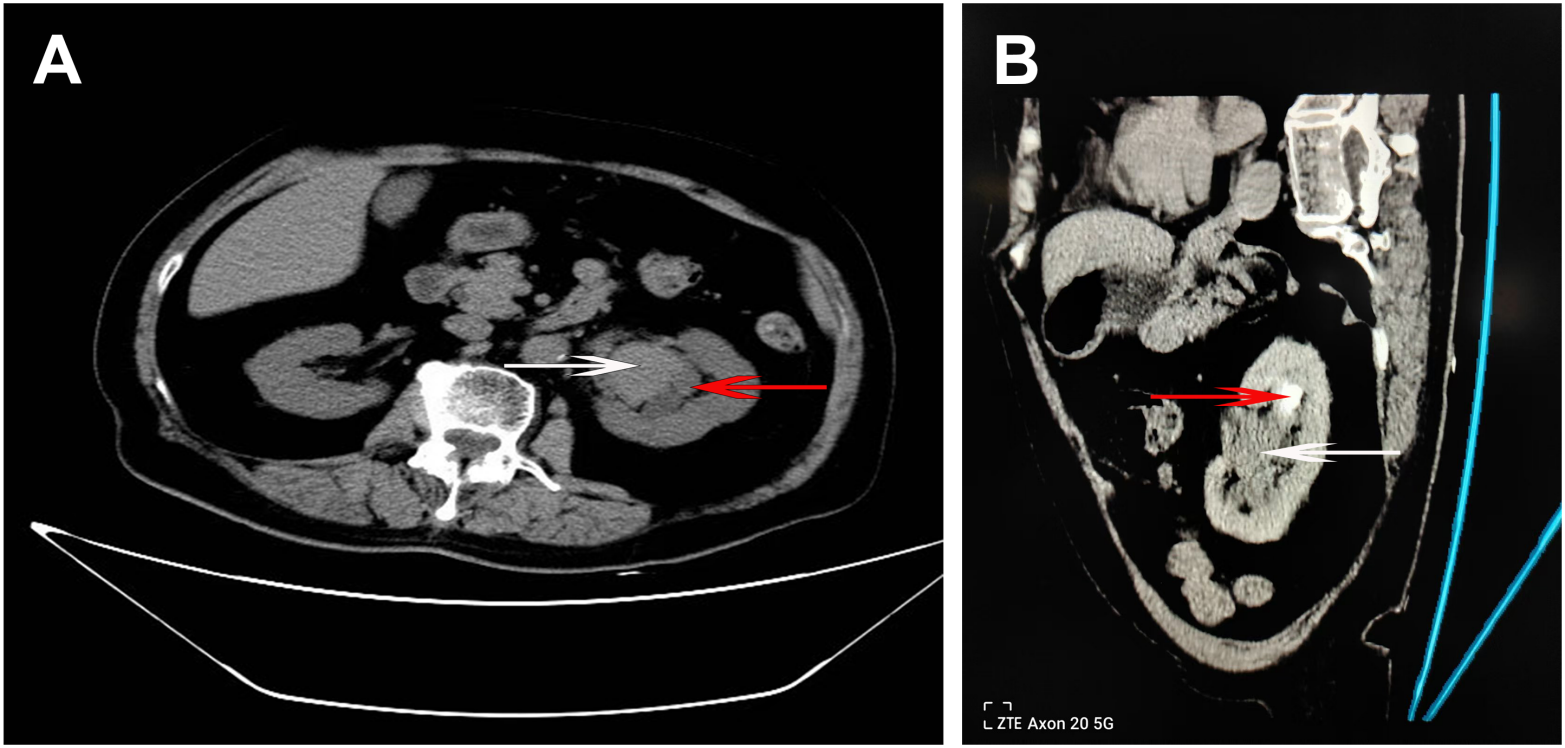
CT imaging of the urinary system. **(A)** Axial non-enhanced CT image shows the primary tumor (white arrow) with mild hyper-density located within the renal pelvis, causing compression and dilation of the adjacent renal calyx (red arrow). **(B)** Oblique enhanced CT image in the late excretory phase reveals the tumor mass (white arrow) within the renal pelvis, accompanied by a filling defect in the upper pole calyx (red arrow) suggestive of possible tumor extension.

A 75-year-old female patient presented with lower abdominal pain and gross hematuria lasting for over 1 month. Subsequently, a non-contrast and enhanced CT scan of the urinary system was conducted and a soft tissue mass located in the left renal pelvis was identified (**Figure 1**).

**Figure 2:**
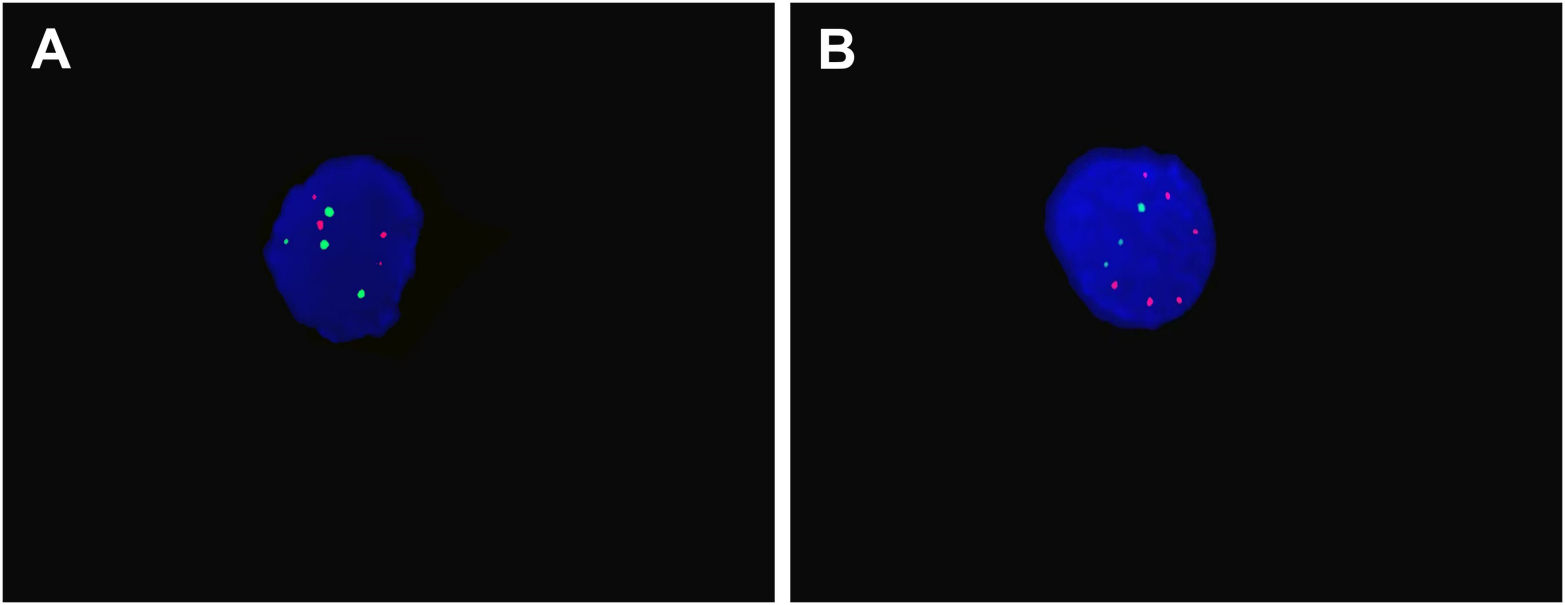
Representative urine FISH results. **(A)** The nucellus shows four red and four green signals for Chromosomes 3 and 7, indicating tetrasomy. **(B)** The nucellus show six red and three green signals for Chromosomes 17 and P16 gene, indicating chromosomal polyploidization and P16 amplification.

Furthermore, FISH assay was performed on urine cytology specimen. As shown in **Figure 2**, polysomic signals of chromosomes 3, 7, and 17 (CEP3, CEP7, CEP17) were observed, indicating significant chromosomal numerical abnormalities. While no deletion was observed at the 9p21 locus (P16 gene), this locus exhibited a remarkable amplification ratio reaching 90%. Based on the above clinical presentation, radiology information and cytogenetic findings, the initial diagnosis was highly suspicious for UC.

## Pathological findings and molecular studies

**Figure 3:**
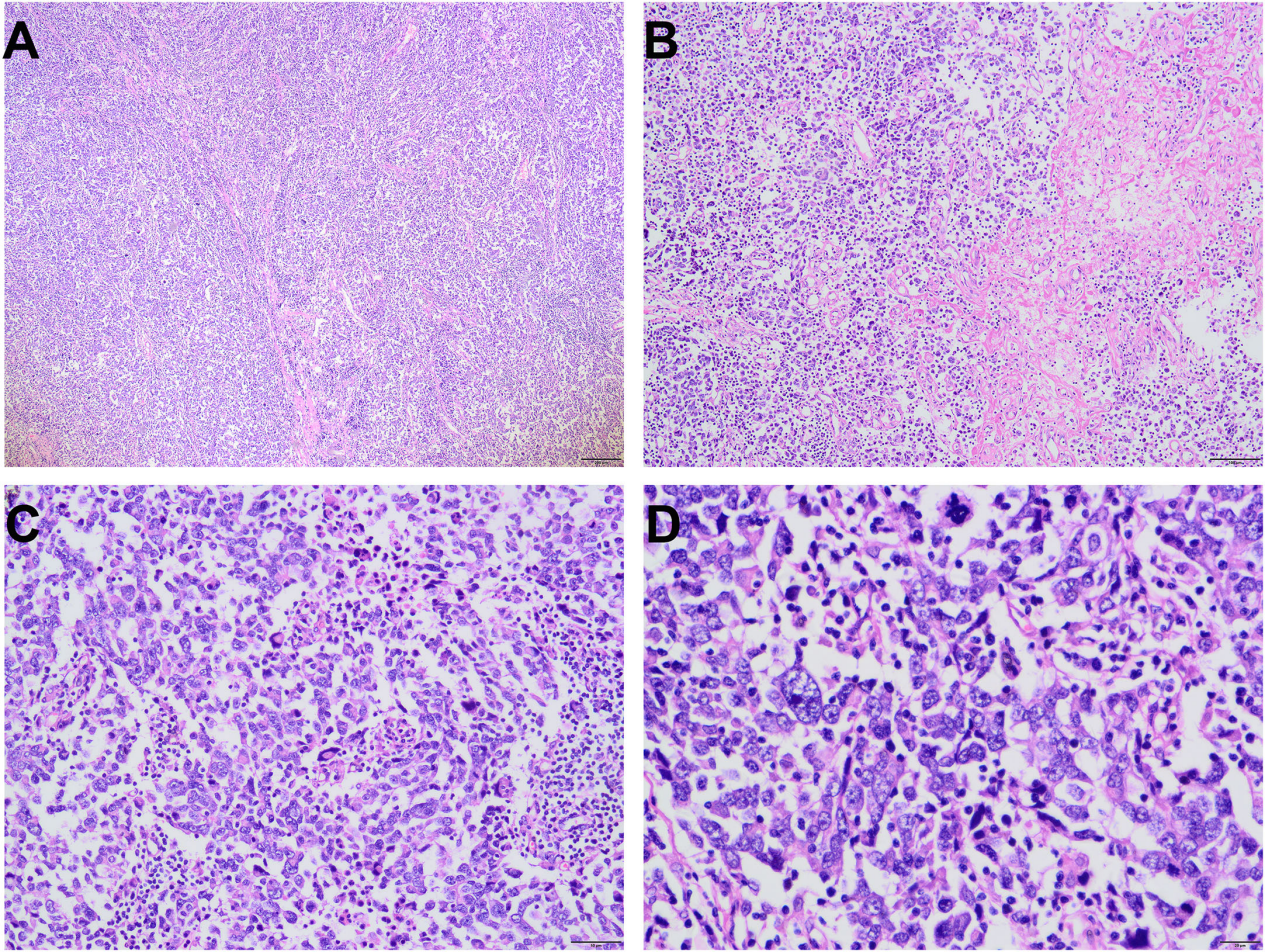
Histopatholgical features of UPS. Tumor cells exhibit solid growth with visible fibrous septa and relatively high cell density and visible necrosis were observed (**(A)**, 40× and **(B)**, 100×). Tumor cells had marked cellular atypia with numerous pathological mitotic figures and visible giant tumor cells [**(C)**, 200× and **(D)**, 400×].

Subsequently, the patient underwent left nephroureterectomy including kidney, ureter, and partial bladder resection. Gross pathological examination of the resected specimen identified a grayish-white to grayish-brown, moderately firm mass measuring approximately 4.8×4.5 cm^2^ located in the renal pelvis. Under the microscope, numerous pleomorphic cells with marked atypia are visible, exhibiting vacuolated nuclei with visible nucleoli; tumor giant cells are relatively common. Pathological mitotic figures are present, accompanied by inflammatory cell infiltration. Focal areas show hemorrhage and necrosis (**Figure 3**).

**Figure 4:**
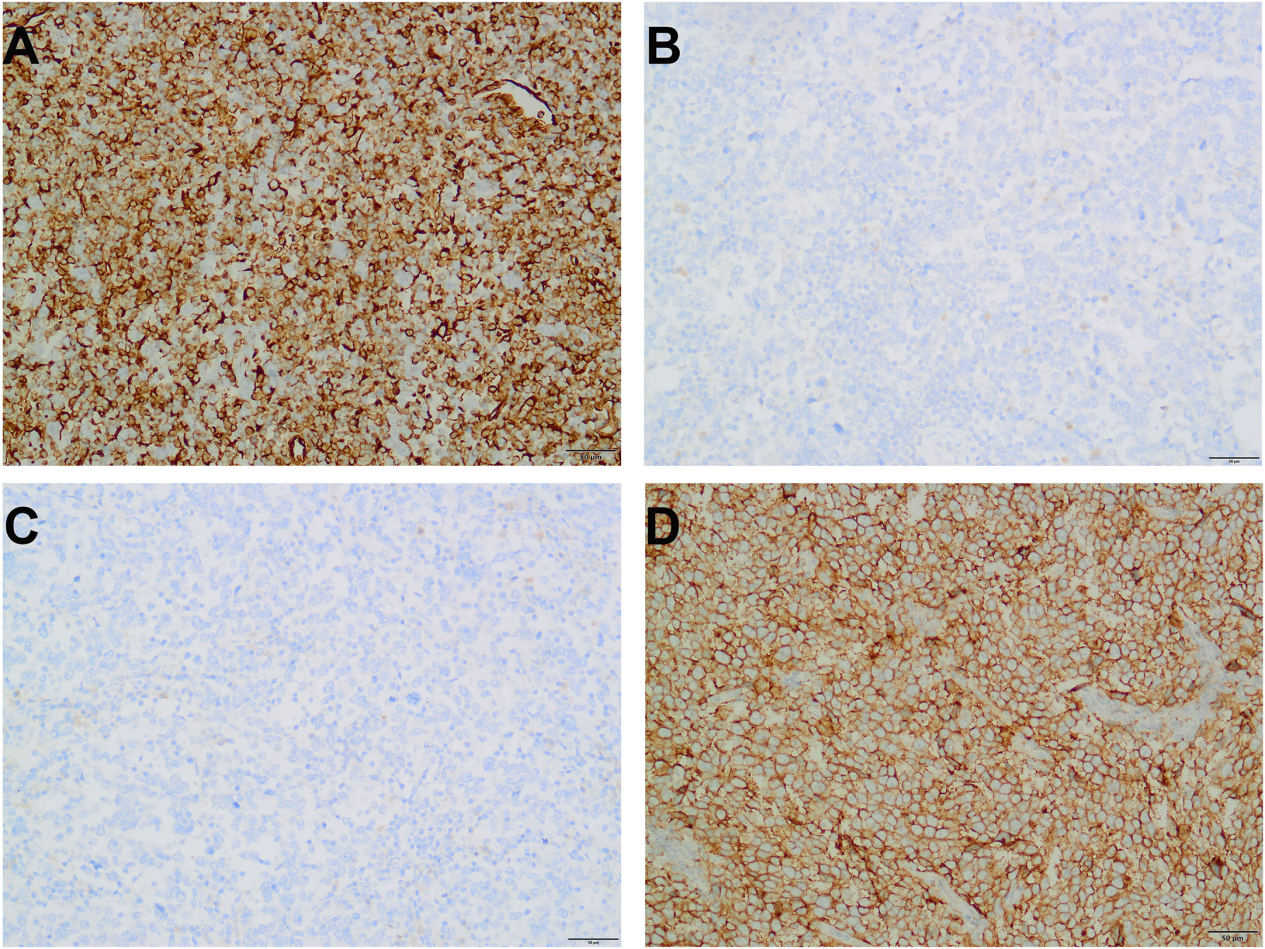
Representative immunohistochemistry results (magnification, 200×). The tumor cells show strong and diffuse cytoplasmic positivity for vimentin **(A)**. Epithelial markers, such as CK **(B)** and EMA **(C)** were negative. PD-L1 is diffusely strongly positive, complete linear membrane staining in 95% of viable tumor cells. No significant stromal or immune cell staining is observed **(D)**.

Immunohistochemical staining was diffusely positive for vimentin and partially for ALK, while the following markers were all negative: CK, EMA, CK5/6, CK20, P63, CK7, GATA-3, CD31, D2-40, CD20, CD79α, CD3, CD30, CD138, S-100, Desmin, CD43, CD2, CD4, and PAX-8 (**Figures 4A–C**). However, no ALK gene rearrangement was identified by FISH analysis. Comprehensive DNA-RNA based NGS profiling analysis revealed no clinically and diagnostic significant gene rearrangements. However, 27 somatic mutations were identified, including a missense variant of certain significance in TP53 p.Lys132Asn (c.396C>A) of exon 5 and a relatively high tumor mutation burden (TMB-H) (**Supplementary Figure 1**).

Subsequent PD-L1 immunohistochemical staining demonstrated strong positivity in the majority of the tumor cells, with a positivity rate of approximately 95%, and a Combined Positive Score (CPS) of 95 (**Figure 4D**). Based on these findings, the patient received intravenous bevacizumab therapy for over a year. To date (28 months post-surgery), the patient has experienced no further discomfort. Regarding imaging examinations, a CT scan performed at our hospital six months after discontinuing medication revealed no evidence of recurrence or metastasis.

## Discussion

UPS is a high-grade soft tissue sarcoma characterized by local recurrence and distant metastasis. Primary renal UPS is uncommon, and cases originating in the renal pelvis are exceptionally rare. In the present case, both radiological and macroscopical examinations confirmed a renal pelvic origin. Histopathological characteristics revealed a high-grade sarcoma with marked pleomorphism and absence of specific differentiation. A large panel of immunohistochemical antibodies and DNA-RNA based NGS were used to exclude other renal entities including specific sarcomas and carcinomas. The tumor cells were negative for epithelial, myogenic, melanocytic, and lymphoid markers, while positive for vimentin and ALK. Therefore, UC and lymphoma were excluded through extensive tissue sampling and the above immunohistochemical markers. Although genetic rearrangements such as BCOR-CCNB3 (4), SS18-POU5F1 (5), and CIC-LEUTX rearrangement (6) were reported in renal sarcomas, no such alterations were identified here by DNA-RNA based NGS, leading to a diagnosis of primary renal UPS.

Urinary FISH is a high sensitivity and specificity assay used to diagnose UC. It detects polysomy of chromosomes 3, 7 and 17, and loss of 9p21 loci of malignant urothelial cells, which are markers of genomic instability. However, Reid-Nicholson et al. observed that these chromosomal abnormalities may also be seen in non-urothelial tumors including adenocarcinoma and squamous carcinoma, especially in primary and secondary adenocarcinoma of the bladder (7). In addition to primary non-UCs, metastatic bladder cancers secondary to other tumors may also yield positive urinary FISH results, such as rectal adenocarcinoma (8) and esophageal cancer (9). Furthermore, urinary FISH positivity may also be observed in certain rare diseases affecting the bladder, such as urachal carcinoma (10), bladder paraganglioma (11), and small cell carcinoma (12). These cases were interpreted as being ‘false’-positive, which has significant implications for the accurate diagnosis, monitoring and management of patients with UC. Beyond these epithelial malignancies, our case provides the first evidence that mesenchymal-derived sarcoma, arising from the renal pelvis, may also yield positive urinary cytology FISH results. Genomic instability, characterized by an increased frequency of genomic mutations, has been highly associated with malignant tumor initiation and progression (13–15). Recently, Ke et al. confirmed that positive urine FISH assays can be found in patients with metastatic tumors and other rare and highly malignant tumors (15). In the present case, NGS analysis identified 27 somatic mutations, including a missense variant in TP53, and revealed a relatively high tumor mutation burden (TMB-H). When the number of tumor cells shed into urine with chromosomal abnormalities reaches the detection threshold, urinary FISH can be positive, which is a marker of genomic instability.

Due to the extreme rarity of primary UPS in the renal pelvis, no standardized treatment protocol currently exists. Surgical resection remains the primary treatment modality for sarcomas, while the necessity of adjuvant radiotherapy or chemotherapy requires further investigation. In addition to these conventional therapeutic approaches, we also try to consider immunotherapy, an emerging treatment modality. PD-L1, a cell surface protein, inhibits T-cell attack by binding to PD1 antibodies on T-cells. Inhibitors targeting this binding pathway can block PD-1/PD-L1 interaction to achieve cancer cell killing. This pathway has been demonstrated to play a role in the development of multiple tumors, including various sarcomas (16). Recent studies have reported PD-L1 overexpression in certain sarcomas, such as UPS (17), alveolar soft part sarcoma (18) and spinal cord sarcoma (19). Additionally, PD-L1 overexpression has been reported in 23-40% of UPS (20, 21). Notably, these reported cases predominantly originate from classic anatomical sites for UPS, such as the limbs, hip joints, pelvis, or head and neck region, rather than the exceptionally rare renal pelvic origin observed in our case. Consequently, additional PD-L1 immunohistochemistry was performed, revealing strong PD-L1expression. Based on this finding, the patient was treated with intravenous Bevacizumab therapy and has since remained free of recurrence or metastasis. Herein, we report the first case of primary UPS occurring in the renal pelvis with PD-L1 strongly positive and validate its potential immunotherapy.

In conclusion, primary renal pelvic UPS is exceptionally rare, which makes diagnosis somewhat challenging. Nevertheless, its distinctive clinicopathological and molecular features can provide profound insights for managing of genitourinary malignancies. Three unique tumor features were identified in this case: distinctive pathological features, the potential for urinary FISH positivity, and PD-L1 overexpression. These underscore the importance of diligently accumulating cases in daily practice and conducting further research to establish diagnostic, therapeutic, and prognostic guidelines for UPS originating in the renal pelvis. Particularly with a positive FISH result, vigilance for a false positive is warranted rather than directly diagnosing it as an UC. Although diffuse positivity for PD-L1 is infrequent in such cases, it is necessary for performing the corresponding test. Given the poor prognosis, precision immunotherapy can be a viable strategy.

## Data Availability

The datasets presented in this study can be found in online repositories. The names of the repository/repositories and accession number(s) can be found in the article/**Supplementary Material**.

## Supplementary material

Supplementary Figure 1 FISH analysis indicates no ALK gene rearrangement **(A)**. NGS testing revealed the presence of TP53 mutation **(B)**.
