# Supplementary figures and images for: Case Report: A rare case of primary undifferentiated pleomorphic sarcoma of the renal pelvis with high PD-L1 expression and a misleading positive urine FISH
Source: Front Immunol. 2026 Mar 3;17:1769769. doi: 10.3389/fimmu.2026.1769769 (PMC12991974; doi:10.3389/fimmu.2026.1769769)

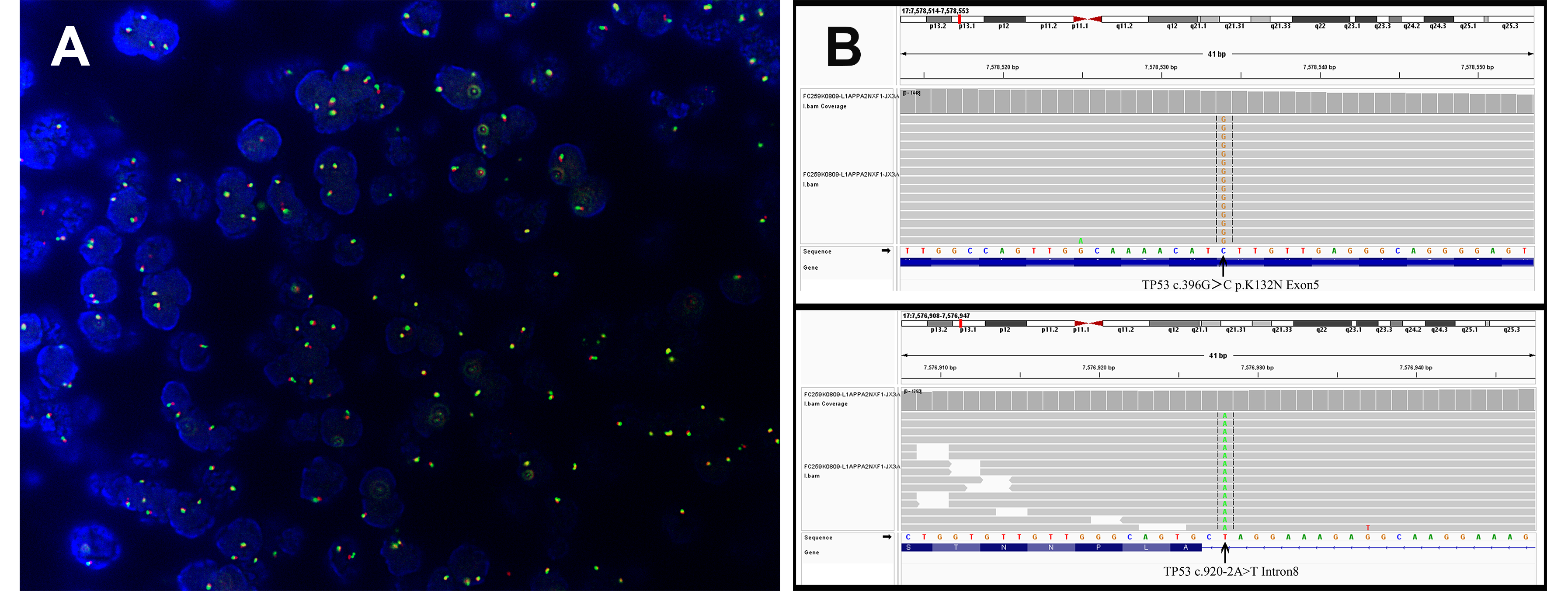

Supplement: Supplementary Figure 1 — FISH analysis indicates no ALK gene rearrangement (A). NGS testing revealed the presence of TP53 mutation (B). [file Image1.tif]
